# The joint evolutionary histories of *Wolbachia *and mitochondria in *Hypolimnas bolina*

**DOI:** 10.1186/1471-2148-9-64

**Published:** 2009-03-24

**Authors:** Sylvain Charlat, Anne Duplouy, Emily A Hornett, Emily A Dyson, Neil Davies, George K Roderick, Nina Wedell, Gregory DD Hurst

**Affiliations:** 1 CNRS (UMR 5558), University of Lyon 1, Laboratoire de Biometrie et Biologie Evolutive, Batiment Mendel, 69622 Villeurbanne, France; 2 The University of Queensland, School of Integrative Biology, QLD 4072, Australia; 3 University of Liverpool, School of Biological Sciences, L69 7ZB, Liverpool, UK; 4 Department of Biology, University College London, 4 Stephenson Way, London, NW1 2HE, UK; 5 University of California Berkeley, Gump South Pacific Research Station, 98728 Moorea, French Polynesia; 6 School of Biosciences, University of Exeter, Cornwall Campus, Penryn, TR10 9EZ, UK

## Abstract

**Background:**

The interaction between the Blue Moon butterfly, *Hypolimnas bolina*, and *Wolbachia *has attracted interest because of the high prevalence of male-killing achieved within the species, the ecological consequences of this high prevalence, the intensity of selection on the host to suppress the infection, and the presence of multiple *Wolbachia *infections inducing different phenotypes. We examined diversity in the co-inherited marker, mtDNA, and the partitioning of this between individuals of different infection status, as a means to investigate the population biology and evolutionary history of the *Wolbachia *infections.

**Results:**

Part of the mitochondrial COI gene was sequenced from 298 individuals of known infection status revealing ten different haplotypes. Despite very strong biological evidence that the sample represents a single species, the ten haplotypes did not fall within a monophyletic clade within the *Hypolimnas *genus, with one haplotype differing by 5% from the other nine. There were strong associations between infection status and mtDNA haplotype. The presence of *w*Bol1 infection in association with strongly divergent haplotypes prompted closer examination of *w*Bol1 genetic variation. This revealed the existence of two cryptic subtypes, *w*Bol1a and *w*Bol1b. The *w*Bol1a infection, by far the most common, was in strict association with the single divergent mtDNA haplotype. The *w*Bol1b infection was found with two haplotypes that were also observed in uninfected specimens. Finally, the *w*Bol2 infection was associated with a large diversity of mtDNA haplotypes, most often shared with uninfected sympatric butterflies.

**Conclusion:**

This data overall supports the hypothesis that high prevalence of male-killing *Wolbachia *(*w*Bol1) in *H. bolina *is associated with very high transmission efficiency rather than regular horizontal transmission. It also suggests this infection has undergone a recent selective sweep and was introduced in this species through introgression. In contrast, the sharing of haplotypes between *w*Bol2-infected and uninfected individuals indicates that this strain is not perfectly transmitted and/or shows a significant level of horizontal transmission.

## Background

Inherited symbionts are an important component of the evolution and ecology of many species. One interaction whose dynamism makes it particularly interesting is that between the Blue Moon butterfly, *Hypolimnas bolina*, and the intracellular bacteria *Wolbachia*. One of the *Wolbachia *strains within this species, *w*Bol1, possesses male-killing ability. Attention was first brought to this strain by the presence of extreme sex ratio bias within certain *H. bolina *populations, with up to 100 adult females to each male [1], associated with 99% of females carrying the *w*Bol1 infection [2]. *H. bolina *lives on islands throughout the Pacific, and as these islands vary in *w*Bol1 frequency, so does their population sex ratio [3]. The variation in population sex ratio affects rates of multiple mating by females, and the size of spermatophore transferred by males during copulation [4].

Variation in the frequency of *w*Bol1 is therefore a very important driver of the reproductive ecology of the butterfly host. The *w*Bol1 strain is also of interest because the extreme prevalence achieved can drive fast evolution in the host, in the form of the spread of suppressor genes that stop the action of the male-killer, and restore the sex ratio to parity [5,6]. When suppressed, the strain *w*Bol1 is maintained by virtue of its ability to induce a second phenotype, cytoplasmic incompatibility, where infected males effectively sterilize uninfected females, driving the infection frequency upward [7]. Accordingly, nearly all females and males recently collected in South East Asia and Samoa are infected with *w*Bol1, and also carry the suppressor of its male-killing activity.

Thus, *w*Bol1 shows spatial variation in both prevalence and phenotype. Indeed, there are also some islands where *w*Bol1 is absent. In the majority of these islands, a distinct infection, *w*Bol2, is present. This strain causes cytoplasmic incompatibility; males carrying *w*Bol2 are incompatible with both uninfected and *w*Bol1-infected females. This incompatibility makes *w*Bol2-infected populations resistant to *w*Bol1 invasion. The *w*Bol2 infection also affects the pattern of gene flow within the species, producing unidirectional reproductive isolation (male immigrants to islands that carry *w*Bol2 do not contribute nuclear genes unless the recipient population also carries *w*Bol2) [8].

The interactions between *H. bolina *and its resident infections therefore represent a particularly interesting case study of the ecology and evolution of *Wolbachia*-host interactions. However, we have a rather poor understanding as to why this system is the way it is. One of the outstanding questions is what allows *w*Bol1 to achieve its extreme prevalence. Theory suggests that for male-killing infections to obtain high prevalence, one of two conditions is necessary (but not sufficient). First, if high rates of horizontal transmission occur in addition to maternal transmission, infection is expected to be present at very high frequency [9]. In the absence of horizontal transmission, standard population genetic models of male-killer dynamics indicate very high vertical transmission efficiency (>99.5%) or a very strong positive impact of infection on the production of host daughters, is required to establish high prevalence [10].

We can investigate these aspects of the *H. bolina *– *Wolbachia *interaction by examining the association between infection status and mtDNA variation. Because intracellular bacteria and mitochondria are co-inherited through the egg cytoplasm, mtDNA can give insight into inherited bacteria transmission patterns, the timing of the spread of an infection, and also the source of infection [11-13]. With respect to transmission patterns, if infections are transmitted with very high efficiency between generations and there is no horizontal transmission between lineages, then the mtDNA of infected and uninfected individuals will evolve to be distinct, because there is little or no flow of mtDNA between infected and uninfected individuals – they represent different subpopulations. In contrast, either inefficient vertical transmission of the bacterium or regular horizontal transmission result in populations of uninfected and infected females becoming homogeneous for their mtDNA diversity [14]. If infection status is a predictor of mtDNA haplotype, this therefore indicates that horizontal transmission is very low, and either vertical transmission rates are very high, or infection is very recent, such that inefficient transmission has not generated uninfected individuals with the 'infected' haplotype. If infection status does not predict mtDNA haplotype, then either there is inefficient vertical transmission or there is horizontal transfer between lineages, or both.

In this study, we obtained partial sequences of the mitochondrial locus CO1 from 298 *H. bolina *individuals of varying infection status, spanning 28 populations. We report on the diversity of mitochondrial haplotypes found, and analyze how mtDNA variation is partitioned by infection status. The structure revealed was surprisingly complex, prompting further exploration of the *Wolbachia *diversity, and the discovery of genetic variation within strains. Overall, these data reject the hypothesis that high prevalence of *w*Bol1 in this system is associated with regular horizontal transmission, and support the hypothesis that high prevalence is associated with very high transmission efficiency. The results also suggest that this infection has undergone a recent selective sweep and was introduced in *H. bolina *through introgression.

## Methods

**Figure 1:**
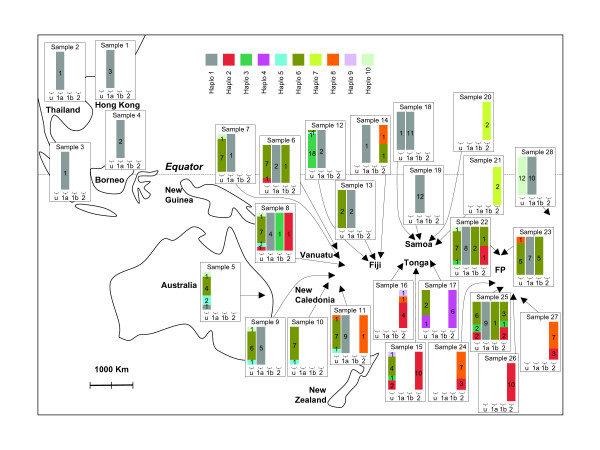
**Geographic location of the populations sampled and distribution of mtDNA haplotypes among islands, partitioned by *Wolbachia *infection status**. Arrow heads indicate geographic origin of the samples. Sample number within each box corresponds to "Sample #" in Table 1. Within each island, the colours within bars indicate the relative proportion of haplotypes within each infection status. Numbers within bars indicate the number of specimens. u = uninfected, 1a = *w*Bol1a, 1b = *w*Bol1b, 2 = *w*Bol2. Details on the distinction between *w*Bol1a and *w*Bol1b are given in the Results section, paragraph five.

Adult *H. bolina *were collected from across the species range during the period June 2000 to April 2006 (Figure 1, Table 1). DNA was extracted and the *Wolbachia *infections assayed by PCR as previously described [3]. To confirm the PCR-based identification, single strand sequences of the *wsp *gene were obtained from 35 specimens following amplification using primer pair 81F-691R [15].

**Table 1:** Description of the samples: location and sample size per sex and infection status

		**Females**				**Males**		
**Island**	**Sample #**	**U**	***w*Bol1**	***w*Bol2**		**U**	***w*Bol1**	***w*Bol2**
**Hong Kong, PR China**	1	0	3	0	0	0	0	0
**Thailand**	2	0	1	0	0	0	0	0
**Peninsular Malaysia**	3	0	1	0	0	0	0	0
**Sabah, Malaysia, Borneo**	4	0	2	0	0	0	0	0
**Australia**	5	6	0	0	0	2	0	0
**Efate**	6	2	3	0	0	6	0	0
**Tanna**	7	2	1	0	0	6	0	0
**Aneityum**	8	5	5	0	0	5	0	1
**Lifou**	9	5	5	0	0	3	0	0
**Grande Terre**	10	4	0	0	0	4	0	0
**Ile des pins**	11	4	9	0	0	5	0	1
**Wayalailai, Fiji**	12	20	2	0	0	0	0	0
**Viti Levu, Fiji**	13	2	2	0	0	0	0	0
**Taveuni, Fiji**	14	0	1	2	0	0	0	0
**Kapa**	15	3	0	5	0	5	0	5
**Neiafu**	16	0	0	3	0	0	0	3
**Niue**	17	3	0	5	0	0	0	1
**Upolu**	18	1	7	0	0	0	4	0
**Savaii**	19	0	7	0	0	0	5	0
**Tutuila, Am. Samoa**	20	0	0	2	0	0	0	0
**Olosega, Am. Samoa**	21	0	0	2	0	0	0	0
**Moorea**	22	5	10	0	0	4	0	2
**Tahiti**	23	1	12	0	0	5	0	0
**Rimatara**	24	0	0	0	0	0	0	10
**Rurutu**	25	5	10	5	0	5	0	1
**Tubuai**	26	0	0	5	0	0	0	5
**Raivavae**	27	0	0	5	0	0	0	5
**Ua Huka**	28	2	8	0	0	10	2	0

(U: Uninfected, *w*Bol1: infected by *w*Bol1, *w*Bol2: infected by *w*Bol2)

Partial sequences of the mitochondrial locus CO1 were obtained from 298 specimens using the primer pair COIf and COIr of Brunton & Hurst [16], yielding a 414 bp sequence. PCR products were sent to Macrogen, Inc. for purification and single strand direct forward sequencing. Any new haplotype was sequenced again using reverse primer to eliminate errors and complete the 5'end of the sequence. Sequences were edited in Sequencher 4.6 (Gene Codes Corporation) and deposited in Genbank (accession nos. AJ844898, AJ844900 – AJ844907). To improve the power of the phylogenetic analysis, sequences of the same locus were obtained from one specimen of each haplotype using the primer pair LCO/HCO [17], yielding a 658 bp sequence.

To assess molecular variation within the *w*Bol1 group in relation with mitochondrial haplotype, we sequenced the five MLST genes of different strains [18], and also utilized the close relatedness of *w*Bol1 to *w*Pip. The *w*Pip strain is polymorphic for a variety of phage element insertion within the genome [19]. We attempted amplification of these elements from *w*Bol1 template using the 16 primers pairs from Duron et al. [19]. Amplification was successful using three pairs (orfs Gp1b, Gp2e, Gp3c).

Differentiation among geographic groups and heterogeneity between the haplotypes of *w*Bol1, *w*Bol2 and uninfected hosts was tested using AMOVA in R [20] using the package seqinR [21].

The phylogenetic relationships amongst the COI *H. bolina *sequences and previously described COI sequences of other *Hypolimnas *species were investigated. Based on previous phylogenetic analysis [22], one species of the genus *Precis *was chosen as outgroup. Phylogenetic analysis was performed using maximum likelihood as implemented in Treefinder [23]. The GTR+G substitution model was chosen following a modeltest analysis [24] as implemented in the HyPhy package [25]. Node support values were calculated using the aLRT (approximate likelihood ratio test) method [26]. To test the hypothesis that the *H. bolina *haplotypes form a monophyletic group, we used Treefinder to compare the likelihood scores of the best tree reconstructed without constraint versus the best tree under the monophyly constraint.

## Results

Partial CO1 sequences were obtained from 298 *H. bolina *specimens, spanning 28 populations (figure 1) and comprising 100 *w*Bol1-infected specimens (89 females and 11 males), 68 *w*Bol2 specimens (34 females and 34 males) and 130 uninfected specimens (70 females and 60 males) (Table 1). Across these 298 sequences, 41 polymorphic sites were observed within the 414 bases sequenced that allowed us to distinguish 10 different sequences. The 10 haplotypes are coded 1 to 10 throughout the text. In addition to PCR-based detection and identification, sequencing of the *wsp *gene confirmed the identity of the infection on a subset of the sample under study (19 *w*Bol1 specimens and 16 *w*Bol2 specimens).

**Figure 2:**
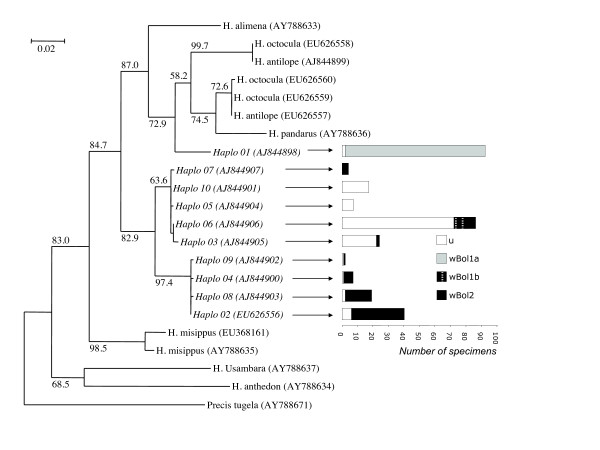
**Phylogeny and distribution of *Wolbachia *infections among haplotypes**. The left part of the figure shows the phylogeny of the 10 *H. bolina *mtDNA haplotypes with respect to *H. alimena, H. octocula, H. antilope, H. pandarus, H. missipus, H. anthedon, H. usambara*, with *Precis tugela *as an outgroup. The tree was constructed via Maximum Likelihood estimation in Treefinder; aLRT (approximate likelihood ratio test) scores are given next to the nodes (only for internal nodes for clarity) and branch length indicates the number of substitution per site. *H. bolina *haplotypes are in italics. Genbank accession numbers are given in parenthesis. The right part of the figure gives the distribution of the various *Wolbachia *infections status (u: uninfected, *w*Bol1a, *w*Bol1b, *w*Bol2) observed with each haplotype. Note the relative proportions of the infections are not representative of the prevalence within the species since sampling was aimed at representing the different infection status in even proportions.

We investigated the phylogenetic relationships amongst the *H. bolina *haplotypes and previously described COI sequences from the same genus. To improve the power of this analysis, longer sequences of the same locus were obtained from one specimen of each haplotype using the primer pair LCO/HCO [17] (except haplotype 7 due to DNA degradation in specimens from American Samoa). The best tree is shown in figure 2. Notably, *H. bolina *haplotypes do not form a monophyletic group in this topology. Whilst haplotypes 2 to 10 do form a monophyletic group, comprising most of the species mtDNA diversity, haplotype 1 falls within another clade also comprising sequences from *H. pandarus*, *H. alimena*, *H. octocula *and *H. antilope*. To further test the hypothesis that *H. bolina *haplotypes form a monophyletic group, we compared the likelihood scores of the best tree reconstructed without constraint versus the best tree under the monophyly constraint. The hypothesis that the constrained tree is the correct tree was rejected (Approximately Unbiased test, p < 0.01). Notably, all the other topology comparison tests implemented in Treefinder (ELW, BP, KH, SH and WSH tests) lead to the same conclusion (p < 0.04). Thus, we conclude that *H. bolina *haplotypes do not form a monophyletic group, suggesting haplotype 1 was introduced in this species through *Wolbachia*-driven introgression.

The genetic distance between *H. bolina *haplotype 1 and the other *H. bolina *haplotypes is considerable by normal standards of intraspecific variation, with around 5% divergence from all other haplotypes. Presence of divergent mtDNA haplotypes within a sample is often regarded as an indicator of the presence of cryptic species. We can reject this hypothesis in the case of *H. bolina*. Males carrying haplotype 1 (associated with the *w*Bol1 infection) are virtually absent from the field, because of male-killing [3]. However, females carrying haplotype 1 are as efficiently fertilised as females carrying haplotype 2–10 [4]. Given that there are no other *Hypolimnas *species present on many of the islands studied (Fiji, Samoa, French Polynesia), this demonstrates that females carrying haplotype 1 (and the *w*Bol1 infection) readily mate in the field with uninfected *H. bolina *males (haplotypes 2 through 10). The 'biological species' is also attested in the laboratory where females of haplotype 1 taken from the field readily produce progeny without further mating, and the subsequent haplotype 1 lineages have been routinely maintained over more than three generations by crossing to sympatric field caught males carrying haplotypes 2 through 10 [3,6,27]. Strikingly, the *w*Bol1 infection is found in association with two very distantly related mitochondrial lineages (Figure 2). Most commonly (90 specimens), *w*Bol1 is found associated with haplotype 1. On rare occasions (10 specimens), haplotypes 3 or 6 (forming a small clade within *H. bolina*) were detected in association with *w*Bol1. Such association with distinct and strongly divergent mitochondria is suggestive of a horizontal transfer of *Wolbachia*, either within *H. bolina *itself, or from an as yet unidentified external source. However, it should be noted that the data also indicates that *w*Bol1 very rarely undergoes horizontal transmission. Horizontal transmission would move *w*Bol1 onto the mtDNA background of any sympatric uninfected individuals. In contrast, *w*Bol1 is absent from the majority of mtDNA haplotypes found in sympatric uninfected *H. bolina *(figure 1).

To further investigate the presence of *w*Bol1 in different mtDNA haplotypes, we sequenced the 5 *Wolbachia *MLST loci from 5 specimens (two bearing haplotype 1 and three bearing haplotype 6). The same sequences (EF589952 to EF589956) were obtained from all specimens, confirming the *w*Bol1 strains present in haplotype 1 and haplotype 6 are closely related, as previously attested by *wsp *sequences. However, neither *wsp *nor MLST sequences are suitable to distinguish between very recently isolated *Wolbachia *lineages. To increase our discriminatory power, we amplified phage WO loci using PCR primers designed for the *w*Pip *Wolbachia *strain [19], which is closely related to *w*Bol1. PCR products were obtained using 3 of the 15 primer pairs. One of these loci (*Gp1b*) showed a clear variation in amplicon size between specimens, with some products being long (~1000 bp estimated from agarose gel) and others short (~330 bp). We obtained sequences from 3 long and 2 short products (Genbank accession numbers FJ215870 and FJ215869). Alignment between sequences was straightforward over the entire length of the short fragment, which corresponds to the 5' region of the long fragment. The two sequences differ by three substitutions across the alignment. We performed the discriminating PCR assay on 57 *w*Bol1-infected specimens. All 48 *w*Bol1-infected individuals associated with haplotype 1 contained the large Gp1b fragment, while individuals associated with the haplotype 3/6 group always contain the small fragment (n = 9). Hence, this PCR assay distinguishes two *w*Bol1 sub-types hereafter called *w*Bol1a (long Gp1b PCR product, strictly associated with mitochondrial haplotype 1) and *w*Bol1b (short Gp1b PCR product, associated with haplotypes 3/6).

The *w*Bol2 infection was found in association with a wide diversity of haplotypes within *H. bolina *(figure 2). Indeed, seven different haplotypes were retrieved from 68 of the *w*Bol2 specimens examined. In addition, no haplotype is private to *w*Bol2-infected specimens, except haplotype 7, which was observed in four specimens.

We used AMOVA to formally assess and test for association between *Wolbachia *infection status and mitochondrial sequence variation. In our dataset, infection status (with four possible levels: uninfected, *w*Bol1a, *w*Bol1b and *w*Bol2) explains 89% of the mtDNA molecular variance (p < 0.001 based on 1000 permutations). Because infection shows geographic structure, we then tested separately for each infection type (*w*Bol1a, *w*Bol1b and *w*Bol2) whether they represent a population significantly differentiated from their sympatric uninfected population, rather than the population as a whole. Only the *w*Bol1b subgroup does not appear to be significantly differentiated from uninfected sympatric populations (*w*Bol1a vs uninfected: p < 0.001; *w*Bol1b vs uninfected: p > 0.38; *w*Bol2 vs uninfected: p < 0.001). This is not surprising given the two haplotypes found in association with *w*Bol1b are also observed in uninfected individuals.

We then examined in detail the evidence for efficient vertical transmission and horizontal transmission rates for *w*Bol1 variants. Efficient vertical transmission and low rates of horizontal transmission would be reflected in differentiation in mtDNA carried by infected and uninfected individuals. We examined differentiation on the 12 islands where *w*Bol1a is found sympatrically with uninfected individuals. All 70 *w*Bol1a individuals on these 12 islands carried haplotype 1. This haplotype was found in only one of 103 sympatric uninfected individuals. Thus, there is clear differentiation between *w*Bol1a-infected individuals and their uninfected sympatric equivalents.

In contrast to *w*Bol1a, both *w*Bol1b and *w*Bol2 exist with more than one mtDNA haplotype, and these haplotypes are shared with uninfected sympatric individuals. Thus, these infections are subject to either inefficient transmission or horizontal transmission or both, and we can gain no definitive information concerning presence/absence of a selective sweep.

## Discussion and Conclusion

We investigated the evolutionary history of *Wolbachia *infections in *H. bolina *using CO1 sequences from 298 specimens. Four general conclusions can be drawn from this analysis, which we will discuss in more detail: (1) *H. bolina *is paraphyletic at the mtDNA level, with up to 5% divergence among haplotypes within what observational and breeding studies indicate is one species; this pattern likely follows from *Wolbachia*-driven introgression; (2) *w*Bol1, the infection competent to kill males, was found with three different mtDNA haplotypes, and examination of *w*Bol1 genetic variation revealed the existence of two cryptic subtypes, *w*Bol1a and *w*Bol1b, *w*Bol1a being found with a single mtDNA haplotype, and *w*Bol1b with the other two; (3) the haplotype observed in *w*Bol1a-infected specimens is extremely rare among sympatric uninfected specimens; in other words, mtDNA allows us to infer that *w*Bol1a has very low levels of effective horizontal transfer, and a very high effective vertical transmission rate (4) no mtDNA variation is found in the haplotypes associated with *w*Bol1a; this pattern is suggestive of a recent selective sweep; in contrast, the *w*Bol2 infection is associated with a large diversity of haplotypes, suggesting it is either ancient and imperfectly transmitted, or shows a significant level of horizontal transmission.

### *H. bolina *is associated with an unusual diversity of mtDNA

There has been an explosion of studies of mtDNA diversity in insects associated with DNA barcoding programmes [28]. These programmes generally find low levels of intraspecific diversity (typically <1%), and high levels of inter-specific diversity (typically >2%). Within the barcoding ethos, there is a 'barcoding gap' between intra and inter-specific variation, and high levels of diversity within a classical species lead to the hypothesis that cryptic species are present. In our case we can be definitive that the *H. bolina *specimens typed for mtDNA are of one species notwithstanding the high level of diversity observed. The evidence base for this derives from a series of observations. First, where male-killing is active, there are few if any sympatric haplotype 1 males with which females can mate in the field, as these are killed by the bacterium. In many islands, *H. bolina *is the only member of the genus present, and thus *H. bolina *haplotype 2–10 males represent the only source of male partners. Second, *w*Bol1-infected individuals carrying haplotype 1 are collected from the field *in copula *with males of haplotypes 2–10. Third, we have routinely maintained *w*Bol1 matrilines carrying haplotype 1 in the laboratory through crosses with sympatric *H. bolina *males of divergent haplotypes.

The breakdown of barcoding as a reliable means of species resolution seen in this case is relatively common in symbiont-infected species [29-31]. It can occur because symbionts drive introgression of mtDNA following hybridization events – the transfer and spread of the symbiont results in spread of the mtDNA from the neighbouring species as observed in *Drosophila *[11,32], and *Acraea *butterflies [12]. This hypothesis would be reflected in paraphyly in a species mtDNA. The best supported tree for mtDNA in the *Hypolimnas *clade does indeed have *H. bolina *mtDNA as paraphyletic, and we can statistically exclude a monophyletic origin of *H. bolina *mtDNA. The fact that the divergent mtDNA lineage is in tight association with the *w*Bol1a strain strongly suggests this pattern results from *Wolbachia *driven-introgression. This interpretation relies on the hypothesis that *H. bolina *males are (or have been in the recent past) capable of producing fertile female offspring following mating with females of other *Hypolimnas *species (belonging to the *H. antilope*/*H. octocula*/*H. pandarus *group), which remains to be verified. While barcoding studies can be a useful first step in species discovery (revealing potentially new cryptic species), caution must be taken to rule out the action of symbiont-driven introgression as an explanation of unusually high mtDNA diversity within a species.

### Mitochondrial variation reveals cryptic *Wolbachia *variation

Sequencing of the *wsp *or MLST genes revealed no molecular variation within the *H. bolina *male-killing *Wolbachia*, *w*Bol1. However, we observed that this infection was found in association with very divergent mtDNA haplotypes (5% distinct). We therefore refined our examination of *w*Bol1 to look for fine scale molecular variation associated with more variable phage insertion loci, as found in detailed studies of *Wolbachia *variation in *Drosophila *and *Culex *[19,33,34]. Variation within *w*Bol1 was observed, and there was a perfect association in our tested specimens between the length of the amplified phage region and mtDNA haplotype. The *w*Bol1a strain, defined by the large fragment, is by far the most frequent in this species, and is found only alongside haplotype 1; *w*Bol1b, defined by the short fragment, is less common, and is found in haplotypes 3 and 6.

What is not clear is how such micro-variation in the mtDNA-*Wolbachia *association arose. If the transmission of *w*Bol1 has been strictly vertical since its establishment in the species, then the mtDNA and *w*Bol1 have both diversified since this time, and the male-killer went extinct in the majority of mtDNA haplotypes. If this hypothesis is correct, then the level of mtDNA divergence between the haplotypes (5%) implies that this infection has been in *H. bolina *for a protracted period. Alternate scenarios rely on horizontal transmission. One hypothesis is that following the arrival of the infection in *H. bolina*, there was a single horizontal transmission event to another mtDNA lineage at some point in the past, moving the infection from one haplotype into the other, followed by fixation of different alleles at the locus in question in the two lineages. An alternative hypothesis is that *w*Bol1 arrived in *H. bolina *at two points in the past, either from one single source or two distinct sources outside the species.

### High prevalence of *w*Bol1a is associated with very high vertical transmission rates

We observed that haplotype 1 was almost private to *w*Bol1a-infected individuals: out of 92 specimens carrying haplotype 1, 90 were infected by *w*Bol1a and only 2 were uninfected. When the data are restricted to the 12 populations where mtDNA sequences were obtained from both *w*Bol1a and uninfected specimens, 70 *w*Bol1a-infected and 1 uninfected specimens carrying haplotype 1 were found, alongside 102 sympatric uninfected individuals of haplotype 2–10.

Had the spread of *w*Bol1a through populations occurred through horizontal transmission, this would be reflected in the presence of *w*Bol1a in the majority if not all sympatric mtDNA haplotypes. That there is a distinct partitioning of mtDNA variation in our study clearly demonstrates that the *w*Bol1a male-killer infection in *H. bolina *shows effectively no ongoing horizontal transmission to individuals of haplotype 2–10, and that vertical transmission is the route by which *w*Bol1a propagates over ecological time. High prevalence can then be achieved in the presence of very high transmission efficiency with low drive (i.e. infected females produce a few more surviving adult daughters than uninfected females), or lower transmission efficiency but with very strong drive (infected females produce many more surviving adult daughters than uninfected females). There is evidence in our data, and in natural history observations, for the former hypothesis. When transmission is inefficient, new uninfected lineages are created that then carry the haplotype of the infected one. If these are viable, then this haplotype will pass into future uninfected individuals and their frequency will build up over time until infected and uninfected lineages carry the same mtDNA profile [14]. Given we know from laboratory studies that uninfected haplotype 1 individuals created by antibiotic treatment are viable and fertile [35], the near absence of this mtDNA haplotype in sympatric uninfected individuals implies either vertical transmission is very efficient, or that the infection is very recent such that there has been insufficient time for the 'drip' of the haplotype 1 to build a significant frequency in the uninfected population. Three facts argue for the former. First, we have yet to observe an 'inefficient transmission' event in the laboratory [27] (Charlat unpublished results). Second, the infection has been present on some of these islands for over 100 years – sufficient time for uninfected individuals of these haplotypes to build up (Hopkins, 1927; Simmonds, 1923; 1926). Third, the extreme prevalence seen on some islands (99% in Samoa) is incompatible with transmission rates below 99.5% [2].

This system is thus compatible with the theory that very high transmission efficiency predisposes to very high prevalence. However, high transmission alone is insufficient to produce high prevalence. The same lack of uninfected females carrying haplotype 1 (that implies very high vertical transmission efficiency) is observed throughout the species range, notwithstanding whether *w*Bol1a prevalence is 25% or 99% [2,8,27]. Thus, we can also infer that perfect vertical transmission does not necessarily produce high prevalence. One possibility is that the equilibrium prevalence is very high, but most populations have not yet attained equilibrium. Alternatively, other and as yet unknown mechanisms might provide the explanation for the variation in prevalence across *H. bolina *populations.

### The contrasted evolutionary histories of *w*Bol1 and *w*Bol2 infections

The results of the present study permit certain inferences to be made about the history of the association between *w*Bol1a and its host. The finding that the *w*Bol1a-infected population exhibits little variation in mtDNA sequence, whilst the uninfected population maintains diversity, suggests that the male-killer infection has undergone a relatively recent selective sweep in this species. Simple absence of diversity in mtDNA in a species can be associated with either a genome wide bottleneck effect or a selective sweep on mtDNA alone. However, comparison between the mtDNA diversity of infected and uninfected individuals allows us to 'internally control' the history of the genome. Where prevalence exceeds 50% of females, the effective population size of infected individuals is greater than that of uninfected individuals. In this case, the neutral expectation is for higher diversity in infected than uninfected individuals. In contrast, haplotypic diversity differs significantly between infected and uninfected individuals, but it is the *w*Bol1a infected population that bears just the single mtDNA haplotype, and its diversity is significantly less than found in the uninfected population. We therefore conclude that the data indicate either a recent spread of the male-killer into the population, or a recent selective sweep of a new strain of an existing male-killer. The system is, in this respect, like that of male-killing *Wolbachia *in both *Acraea encedon *and *Acraea encedana *[12], but in contrast to the equilibrium situation observed in the male-killing *Wolbachia*/*Drosophila innubila *interaction [36].

We can also make some comments concerning the dynamics and history of the *w*Bol2 infection (non-male-killing) within *H. bolina*. The sharing of haplotypes between *w*Bol2-infected individuals and uninfected individuals indicates that this *Wolbachia *is either not perfectly transmitted, or shows a low level of horizontal transmission, in contrast to the male-killing infection. In addition, the finding that the *w*Bol2 infection is associated with a relatively diverse array of mtDNA haplotypes indicates that, with the assumption that horizontal transmission is rare, this infection has not undergone a recent selective sweep and is not a very recent acquisition.

## Authors' contributions

SC designed the study, collected specimens, produced and analysed data, and wrote the manuscript. AD, EH and ED collected specimens, produced data and helped to draft the manuscript. NW, ND and GR helped to design the study and to draft the manuscript. GH instigated and supervised the study, collected specimens, analysed data, and wrote the manuscript. All authors read and approved the final manuscript.
